# Biotransformation of 17β-Estradiol through a Denitrifying Sludge

**DOI:** 10.3390/ijerph192013326

**Published:** 2022-10-15

**Authors:** César Camacho-López, Claudia Romo-Gómez, Elena María Otazo-Sánchez, Otilio Arturo Acevedo-Sandoval, Edelmira García-Nieto, Libertad Juárez-Santacruz

**Affiliations:** 1 Área Académica de Química, Universidad Autónoma del Estado de Hidalgo, Mineral de la Reforma 42184, Hidalgo, Mexico; 2 Centro de Investigación en Genética y Ambiente, Universidad Autónoma de Tlaxcala, Tlaxcala de Xicohténcatl 90000, Tlaxcala, Mexico

**Keywords:** 17β-estradiol, denitrification, biodegradation, emerging pollutants, mineralization

## Abstract

17β-estradiol (E2) is the natural estrogen with the most significant potential for endocrine disruption in the biota of aquatic ecosystems at trace concentrations. It is, therefore, essential to study treatments for water polluted with E2 that would guarantee its complete elimination and mineralization. Denitrification is a biological process shown to have the capacity to completely biodegrade drugs, such as ampicillin. This work is aimed to evaluate the biotransformation of 17β-estradiol by employing a denitrifying sludge. The assays performed were: (I) abiotic with 3.5 mg E2-C L^−1^ and (II) denitrifying with 10 mg CH_3_COO^−^-C L^−1^ as the reference, 10 mg E2-C L^−1^ as the sole electron donor, and a mixture of (mg L^−1^) 10 E2-C with 10 CH_3_COO^−^-C at C N^−1^ of 1.1. The E2-C and NO_3_^−^-N consumption efficiencies were greater than 99%, and HCO_3_^−^-C and N_2_ production yields were close to 1 in all assays. The denitrifying sludge could biodegrade up to 10 mg E2-C L^−1^ as the sole electron donor and when mixed with 10 mg CH_3_COO^−^-C L^−1^. No intermediate metabolites were generated from the process.

## 1. Introduction

Water is one of the primary natural resources responsible for the development of the different life forms on the planet. Nearly 70% of the surface of the earth is covered with water, of which 2.5% is fresh water. However, less than 1% of the latter is available for human consumption [1]. Exponential population growth has led to the overexploitation of water to cover anthropic requirements. This has contributed to the incorporation and increase in contaminant concentrations in the water, reducing the quality and availability of this resource. The scarce number, or lack, of treatments for domestic and industrial effluents contributes to the pollution of natural water bodies [2], producing the alteration of the physicochemical properties of the water [3]. A pollutant group now attracting the attention of the scientific community is the emerging contaminants (ECs) [4,5]. ECs are a group of organic compounds of different chemical natures used in everyday products; this is why their presence in the environment is not necessarily new [6,7,8]. Currently, there are no regulations for many of these pollutants, or these are not entirely clear. Only some of the ECs are under consideration for future regulations, mainly because of their known effects on human health and environmental safety.

The ECs include industrial and food additives, disinfectants, detergents, drugs, hydrocarbons, solvents, pesticides, personal care products, and flame-retardants, among others [9,10,11]. Studies show that these compounds’ physical and chemical properties produce certain persistence, bioaccumulation, and toxicity, causing irreversible damage to the flora and fauna of aquatic ecosystems [9,12,13].

The presence of ECs in natural water bodies has been increasing, which has generated global concern about their toxic effect on the biosphere. Ebele et al. [6] reported the uptake and depuration of pharmaceuticals in water reclaimed by the mosquito fish (Gambusia holbrooki). Bioaccumulation factors measured for caffeine, diphenhydramine, diltiazem, carbamazepine, and ibuprofen were 2.0, 16, 16, 1.4, and 28, respectively. Hoeger et al. [12] reported that when brown trout (*S. trutta f. fario*) is exposed to diclofenac concentrations of 0.5, 5, and 50 µg L^−1^, histopathological effects were found in kidneys and the liver. These reports indicate that the presence of drugs in water bodies is causing damage to aquatic biota.

17β-estradiol (E2) and its metabolites (estrone (E1), estriol (E3), and ethinylestradiol (EE2)) are hormonal chemical compounds considered drugs that are produced naturally and synthetically [14,15]. They are characterized by an aromatic ring, which has a high affinity for estrogen receptors in humans; two cyclohexane rings; and a pentane ring [16].

Estrogens are widely used as drugs for diseases in humans, in aquaculture, and in veterinary medicine. In the latter case, an average discharge of 83 tons per year has been estimated in the United States and the European Union [17]. Estrogens are not entirely metabolized and are physiologically secreted through the urine [18,19]. Furthermore, a pregnant woman has been shown to secrete up to 5 mg of E2 daily [20].

E2 and its metabolites are considered the largest endocrine disruptors in water at an order of 10,000 to 100,000 times higher than other synthetic compounds, such as organochlorines found in trace concentrations [20]. E2 trace concentrations were reported to induce feminization, reduce fertility, cause oxidative stress, and decrease sperm quality in fish [18,21,22,23].

Ma et al. [24] reported 180, 64, 18, and 42 ng L^−1^ of E1, E2, E3, and EE2, respectively, in effluents from conventional wastewater treatment plants, which indicates the low efficiency of the processes used for their disposal. Hence, among the processes studied to eliminate these compounds (Table 1) are the advanced oxidation processes (AOP). AOPs are believed to be among the most promising treatments for removing E2 and its metabolites. However, they can also be a possible source of by-products that preserve the mode of action of the original compound or that may be even more biologically active [13,25]. For example, Bennett et al. [19] and Orozco et al. [23] reported that E2 removal efficiency was up to 90%, but they did not mention the final products of E2 oxidation. However, they do not mention free-radical formation due to reactions with the problem compound that occurs with oxidizing reagents, such as H_2_O_2_ and UV light (Table 1).

On the other hand, aerobic and anaerobic biological processes have also been studied for E2 elimination (Table 1). However, as with the advanced oxidation processes, transformation products were not reported, the efficiencies were less than 80%, and metabolites, such as estrone (E1), were formed. Moreover, in most cases, they do not mention the possible physical and chemical events occurring during the experiments [21,27].

The denitrifying process was studied in the biotransformation of some recalcitrant contaminants. It was completely efficient in the reduction of NO_3_^−^ to N_2_. Denitrification also mineralized up to 22 mg 2-chlorophenol-C (2-CP) L^−1^, 92 mg phenol-C L^−1^ [28], and 125 mg toluene-C L^−1^ d^−1^ in a mixture with 125 mg CH_3_COO^−^-C L^−1^ d^−1^ [29], 100 mg toluene-C L^−1^, and 70 mg *m*-xylene-C L^−1^ [30], among others. There are few reports on this process using pharmaceutical products. Islas et al. [4] reported the total biotransformation of NO_3_^−^ to N_2_ and 10 mg ampicillin-C (AMP-C) L^−1^ to HCO_3_^−^ and CO_2_. Other studies evaluating the denitrifying process do not report the final destination of the contaminants [31,32]. Likewise, no physical or chemical phenomena are carried out during the experiments. Such is the case of the study by Ruan et al. [32], which used oxytetracycline and ciprofloxacin from 50 to 300 µg L^−1^, where only the decrease in the antibiotic concentrations is evaluated.

Considering the presence of E2 in effluents from conventional wastewater treatment plants and natural water bodies, information is required on the efficiency of the processes and the final destination of the E2 in biological systems such as denitrification. That is why the hypothesis of this work is that the application of the denitrifying process makes possible the biotransformation of E2 to HCO_3_^−^ in water for its application in a larger-scale process. This is an alternative to the mineralization of E2 in wastewater, and it may avoid discharging into natural water bodies. Hence, the aim of this work is to study 17β-estradiol biotransformation, as a sole electron donor and in a mixture with CH_3_COO^−^, through a denitrifying sludge; likewise, we also aim to contribute to the knowledge about mineralization and the final destination of the E2, as well as to evaluate its effect in the respiratory process of the denitrifying sludge through consumption efficiency, production yield, and species-specific rate. The results obtained can be the basis for the scaling up of this process in wastewater treatments containing E2, creating a carbon source of easy biodegradability. This is because the current research mentions that one of the sources of E2 in natural water systems is poor treatment in conventional processes.

## 2. Materials and Methods

### 2.1. Inoculum Source

An up-flow anaerobic reactor (UASB) was installed and operated with the following dimensions: total volume of 1.5 L; internal diameter, external diameter, and height of 8, 8.18, and 53 cm, respectively; with a working volume of 1.34 L. The reactor was inoculated with 4.5 ± 0.004 g volatile suspended solids (VSS) L^−1^ of a methanogenic-origin anaerobic sludge from the Atotonilco de Tula Wastewater Treatment Plant, Hidalgo, Mexico. It was monitored for 136 days. It was operated at a hydraulic retention time (HRT) of 2 days at a temperature of 30 ± 0.01 °C and a pH of 8.15 ± 0.7. The reactor was fed with two independent media to avoid the precipitation of the components. The first media with the carbon source consisted of (mg L^−1^) KH_2_PO_4_ (1000), Na_2_MoO_4_·2H_2_O (120), CaCl_2_·2H_2_O (300), MgSO_4_·7H_2_O (400), and CH_3_COO^−^-C (1000), whereas the second media with the nitrogen source had the following composition (mg L^−1^): FeCl_3_·6H_2_O (100), CuSO_4_·5H_2_O (20), and NO_3_^−^-N (555.55). The denitrifying respiratory process of the sludge in the UASB was evaluated by calculating substrate consumption efficiency (E), product yield (Y), and the volumetric rate of the substrate and product (q), as well as the balance of the mass of carbon and nitrogen species. On reaching the denitrifying steady state (DSS), sludge samples were taken from the UASB reactor. Samples were washed three times with a physiological solution (0.9% NaCl) before using them as an inoculum source in the batch tests. This process was carried out according to a report by Islas et al. [4].

### 2.2. Batch Assay Conditions

Serological bottles of 60 mL with 80% working volume were used for the batch assays. The following composition of the mineral medium was added to each bottle (mg L^−1^): KH_2_PO_4_ (500), Na_2_MoO_4_·2H_2_O (250), CaCl_2_·2H_2_O (150), MgSO_4_·7H_2_O (100), FeCl_3_·6H_2_O (50), and CuSO_4_·5H_2_O (6). The bottles were inoculated with 0.5 gr VSS L^−1^ of the stabilized denitrifying sludge. Each bottle was sealed with a rubber stopper and an aluminum cap. A stream of argon for 5 min was used to displace the air from the headspace of the flask to obtain anoxic conditions. Subsequently, the bottles were incubated at 34 °C and 200 rpm. Each assay was performed in duplicate and discarded after sampling.

### 2.3. Abiotic Control Assays

Two abiotic control assays were carried out. The first assay took place in the absence of the denitrifying sludge, with 3.5 mg E2-C L^−1^ at a C N^−1^ of 1.1. It was used to evaluate the loss of substrates due to chemical reactions and volatilization. The second assay, with the sterilized denitrifying sludge (121 °C for 1 h), added 3.5 mg E2-C L^−1^ to a C N^−1^ of 1.1 to evaluate the losses due to physical events, such as adsorption under denitrifying conditions.

### 2.4. Denitrifying Assays with 17β-Estradiol and Acetate

These assays consisted of (I) 10 mg CH_3_COO^−^-C L^−1^, which was taken as the reference; (II) 10 mg E2-C L^−1^, to evaluate the biodegradation capacity of the denitrifying sludge and the destination of this compound; and (III) a mixture of 10 mg E2-C L^−1^ and 10 mg CH_3_COO^−^-C L^−1^ to evaluate the effect of an easily biodegradable source in the presence of an emerging contaminant. A mineral medium was added to each one under the composition and conditions previously described, at a sufficient NO_3_^−^-N concentration for a C N^−1^ of 1.1.

### 2.5. Analytical Methods

The E2 concentration was determined using high-performance liquid chromatograph (HPLC) (PerkinElmer Series 200, Norwalk, CT, USA), equipped with a PerkinElmer UV–Vis detector, establishing a wavelength (λ) of 230 nm. An IB-Sil column (C8 5 µm, 250 × 4.6 mm) was used. The mobile phase consisted of an acetonitrile/water solution (50:50, *v*/*v*) and a flow rate of 1 mL min^−1^. NO_3_^−^ and NO_2_^−^ were analyzed in a PerkinElmer Series 200 HPLC (USA) equipped with a PerkinElmer UV–Vis detector, establishing a wavelength (λ) of 214 nm. The anionic column used was a Dionex IonPac AS11-HC-4µm Capillary Column (0.4 × 250 mm). The mobile-phase composition (mL) consisted of water (840), acetonitrile (120), butanol (20), and borate gluconate solution (20). The borate gluconate solution consisted of sodium gluconate (16), boric acid (18), sodium tetraborate decahydrate (25), and 25% glycerol at a volume of 1 L. The mobile-phase flow was set a 1 mL min^−1^. CH_3_COO^−^ was determined using a gas chromatograph (GC) (PerkinElmer AutoSystem XL, Norwalk, CT, USA) equipped with a flame ionization detector (FID) and a capillary column (Agilent DB-1701 USA) of 30 m × 0.25 mm × 0.25 µm. The injector, oven, and detector temperatures were 230, 180, and 260 °C, respectively. The gas composition (N_2_, N_2_O, and CO_2_) in the headspace was determined by a PerkinElmer AutoSystem XL GC (USA) equipped with a thermal conductivity detector (TCD) and a capillary column (Agilent HP-Plot/Q USA) of 15 m × 0.53 mm × 0.40 µm. The injector, oven, and detector working temperatures were 100, 50, and 110 °C, respectively. Argon was used as the carrier gas at a flow rate of 10 mL min^−1^. The HCO_3_^−^ was determined in the form of CO_2_ using the same GC (PerkinElmer AutoSystem XL, Norwalk, CT, USA) under the abovementioned conditions. The sample preparation consisted of 2 mL of the sample and adding 300 µL of HCL (38%) to lower the sample pH and break the equilibrium, transforming all the HCO_3_^−^ into CO_2_. Total, fixed, and volatile suspended solids (TSS, FSS, VSS) were determined according to standard methods [33].

### 2.6. Response Variables

The sludge kinetics in the batch assays were evaluated by calculating the consumption-specific rate (q) of substrates and the generation of products (q) (mg substrate or products g VSS^−1^ h^−1^). The specific rates were determined through a linear regression analysis of substrate consumption and product generation profiles (R^2^ > 0.9). The respiratory process of the denitrifying sludge was evaluated by calculating substrate consumption efficiencies (E) ([mg substrate consumed/mg substrate fed] × 100) and product yields (Y) (mg product generated/mg substrate consumed).

### 2.7. Statistical Data Processing

Calibration curves were performed in triplicate for each chemical species. In all cases, the determination coefficients (R^2^) were greater than 0.98, and the variation coefficients in the analysis of the compounds involved in the assays were less than 10%. Results represent the mean and standard deviations of at least two independent trials. A two-way variance analysis (ANOVA) was performed with the results of the response variables obtained from the reference assay, with E2 and the mixture of E2 and CH_3_COO^−^ (*p* > 0.05) used to find differences between the results obtained. Likewise, a multiple comparison test (Tukey) (α = 0.05) was performed.

## 3. Results and Discussion

In the UASB reactor, a denitrifying steady-state sludge was obtained. It served as the inoculum in the abiotic batch assays and with the activated sludge. The abiotic results of the assays with E2 and NO_3_^−^, in the absence and presence of sterilized denitrifying sludge, showed that after 144 h of incubation, the substrate concentrations decreased in all assays. However, the presence of new chemical compounds in the aqueous medium or the headspace was not observed in the chromatograms. This shows that the substrates do not react between themselves and the medium; they do not volatilize or absorb but precipitate under denitrifying conditions. Finally, the respiratory process of the sludge was evaluated for the biodegradation of CH_3_COO^−^-C and 17β-estradiol as the electron sources. With the results obtained, the metabolism and kinetics of the process were analyzed.

### 3.1. Biodegradation of Acetate and Nitrate in the UASB Reactor

The results from the UASB reactor showed that the DSS was reached after 100 days of operation. Production yields (Y) of HCO_3_^−^-C and N_2_ were greater than 0.9, with a biomass yield (Y_Biomass_) of 0.09 ± 0.01. Consumption efficiencies of CH_3_COO^−^-C (E_CH3COO-C_) and NO_3_^−^-N (E_NO3-N_) were greater than 98%.

### 3.2. Abiotic Evaluation Assays

The results obtained from the abiotic tests carried out with the mineral medium, both with 3.5 mg E2-C L^−1^ and 3.18 mg NO_3_^−^-N L^−1^, in the absence and presence of the sterilized denitrifying sludge, indicated that there was a decrease of 53 and 48% in E2-C and NO_3_^−^-N in the first 24 h, respectively. After 144 h of reaction, 44 and 71.38% of the substrates were quantified at the end of the assays. The behavior in the abiotic assays was not associated with chemical reactions or the volatilization of the substrates. This is because the presence of new chemical compounds in the aqueous medium or the headspace was not observed in the chromatograms. It is worth mentioning that turbidity was present, due to the constituents, at the beginning and at the end of the assay in the system. Therefore, the substrates were not completely solubilized, showing that the difference in the initial concentrations was directly attributed to the precipitation of the substrates.

The solubility of organic chemical compounds depends on temperature, pH, and ionic strength [34,35,36]. In this sense, Shareef et al. [37] report that pH significantly affects E2 solubility, so that at pH values lower than pKa (10.46), E2 is in its neutral form, and it is more difficult to solubilize. Given the working conditions, at which the pH was 7.0 ± 0.01, it was considered that the pH contributed to the partial precipitation of the initial concentration, leaving a remainder of 1.89 mg E2 L^−1^ until the end of the assay (144 h). Li et al. [27] reported that when they studied, in abiotic assays, the interactions of 1 mg L^−1^ E2 with *N. europea*, which were previously sterilized in a nitrifying process, the concentration remained constant throughout the reaction time (192 h). This indicates that if the concentration of E2 is reduced to 1 mg L^−1^, it is unlikely that precipitation would occur.

### 3.3. Denitrifying Reference Assays

**Figure 1:**
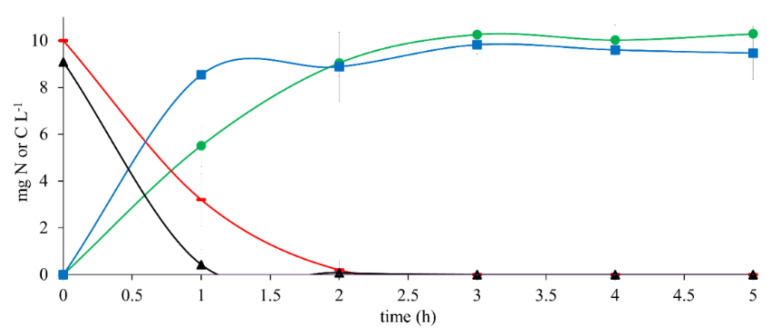
Kinetic profile of substrate consumption and product generation in denitrifying reference assays with 10 mg CH_3_COO^−^-C L^−1^ at C N^−1^ of 1.1. (**▬**) CH_3_COO^−^-C, (▲) NO_3_^−^-N, (●) HCO_3_^−^-C, (■) N_2_.

The results of the reference assay with 10 mg CH_3_COO^−^-C L^−1^ as the sole electron donor (Figure 1) showed an E_CH3COO_^−^-_C_ and E_NO3_-_N_ of 100% after a 2 h reaction time. Simultaneously, HCO_3_^−^-C and N_2_ were produced with Y_HCO3_-_-C_ of 1.01 ± 0.01 and Y_N2_ of 1.12 ± 0.01 at the end of the assay. The results of q_CH3COO_-_-C_ and q_NO3_-_-N_ were 9.79 ± 1.09 and 9.01 ± 2.40, respectively. The q_HCO3_-_-C_ and q_N2_ obtained from this trial were 6.86 ± 0.68 and 6.88 ± 0.86, respectively (Table 2).

These results indicated that the denitrifying consortium performance was efficient and complete. It was used as the reference for assays with 17β-estradiol and in a mixture with CH_3_COO^−^.

### 3.4. Biodegradation of 17β-Estradiol as the Sole Electron Donor

**Figure 2:**
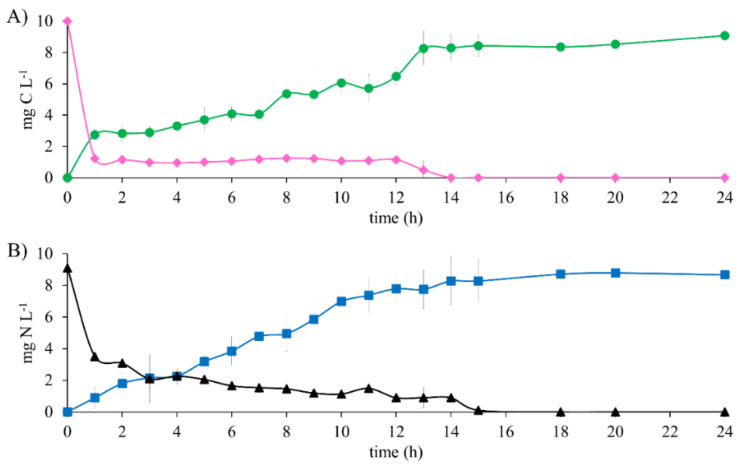
Substrate consumption profile and generation of denitrifying assay products with 10 mg E2-C L^−1^ at C N^−1^ of 1.1. (**A**) Carbon compounds: (♦) E2-C, (●) HCO_3_^−^-C. (**B**) Nitrogen compounds: (▲) NO_3_^−^-N, (■) N_2_.

The results of the metabolic evaluation of the denitrifying sludge show the total substrate consumption after 15 h of reaction time in the biodegradation of 10 mg E2-C L^−1^ as the sole electron source (Figure 2). An E_E2-C_ and E_NO3_-_-N_ of 100% were obtained. However, the HCO_3_^−^-C production analysis was not equivalent to E2 consumption during the first 12 h of the reaction. This was because E2-C decreased 87.6% after 1 h and only produced 27.41% of HCO_3_^−^-C. This is justified by the results obtained in the abiotic tests. There was partial precipitation of the substrates. This comparison made it possible to establish that the actual consumption percentage of E2 was 27.41% and that 60.19% precipitated.

It is noteworthy that, as the test progressed, the E2 that was insoluble at the beginning of the test gradually became soluble after 12 h of the reaction. Hence, the consumption profile of the substrate shows that from 1 h to 12 h of reaction time, there is a constant concentration (Figure 2A). In contrast, the same profile shows that after 12 h, there is a constant decrease in the concentration until it was exhausted at 15 h. Similar behavior was observed with NO_3_^−^; both substrates were consumed at 15 h. After this time, the concentrations of HCO_3_^−^-C and N_2_ were constant until the end of the test, which lasted 24 h. This study shows clear evidence of the complete biodegradation of E2 to HCO_3_^−^ and NO_3_^−^ to N_2_, obtaining a Y_HCO3_-_-C_ and Y_N2_ of 0.90 ± 0.03 and 0.95 ± 0.02, respectively. After performing an analysis of the means of the results obtained of the efficiency and yield in the reference assays with CH_3_COO^−^-C as the electron source and those obtained with E2-C, no statistically significant differences were found. The kinetic profile (Figure 1 and Figure 2) shows that in the presence of E2, the NO3-consumption time is greater than in the reference assays by 86%. Nonetheless, the denitrifying respiratory process is carried out effectively. It was able to show that the denitrifying sludge could consume and mineralize 10 mg E2-C L^−1^, according to the mass balance (Table 3). It is noteworthy that, during the assays, the carbon and nitrogen intermediaries were not recorded.

In the present study and under the conditions described above, the performance of the denitrifying sludge showed better results than the study reported by Li et al. [17]. They mentioned that after 168 h of reaction time, they managed to eliminate 96% of 15.8 mg L^−1^ E2-C in the presence of 0.1 mg L^−1^ of tetracycline by *Novosphingobium* sp. Although they report a high percentage of estradiol elimination, they do not mention the final destination of the contaminant. Hence, they just evaluated *Novosphingobium* sp. growth, assuming that the decrease in the concentration of E2 was used for cell growth. These authors also report a decrease in the number of bacterial cells, which contradicts the previous argument. The results obtained in the current study are promising, given that 100% efficiency was obtained in a period that is 11 times less than that reported by Li et al. [17]. Biodegradation and the effects of 2 mg of E2 L^−1^ were evaluated by Li et al. [26] using *S. dimorphus*. They reported that the presence of E2 affected the growth and photosynthetic activity of *S. dimorphus*, attributing it to a 46.1% decrease in the E2 concentration. However, they do not mention the evaluation of physical and chemical processes that could occur during the assays. It is impossible to associate a 46.1% decrease in the concentration exclusively with the growth of *S. dimorphus.*

It is noteworthy that, although there was an effect of precipitation in the first 12 h of the reaction in this study, the total and simultaneous consumption of E2-C and NO_3_^−^-N was exclusively attributed to the denitrifying respiratory process. On the other hand, Fernández et al. [21] and Xiong et al. [14] evaluated the elimination of 3.9 and 7.9 mg E2-C L^−1^ in anoxic conditions with *Bacillus licheniformis* and *Deinococcus actinosclerus* strains, respectively; these studies show a consumption of E2 of 68 and 90% in 90 and 5 days, respectively. Both studies showed a long reaction time and the generation of secondary metabolites such as estrone (E1). In contrast, in this study, a concentration of 10 mg E2-C L^−1^ was evaluated, which was 61 and 21% higher than the values found by Fernández et al. [21] and Xiong et al. [14], respectively. The total E2 consumption was carried out in just 15 h, and the formation of secondary metabolites was not recorded. Likewise, E2 and NO_3_^−^ were fully mineralized to HCO_3_^−^ and N_2_, respectively (Table 3).

Haiyan et al. [18] conducted a study with 24 mg L^−1^ of 17α-ethinylestradiol-C using *Sphingobacterium sp*. under aerobic conditions, where they reported an 87% degradation in 240 h of reaction time. The study identified 2-hydroxy-2,4-dienevaleric acid and 2-hydroxy-2,4-diene-1,6-dioic acid as the end biodegradation products. Therefore, the authors suggest further studies to achieve the complete mineralization of this compound. Moreover, the advanced oxidation processes (AOPs) were studied to eliminate 7.9 µg L^−1^ E2-C; this study reported less than 85% efficiencies. Reports show that some free radicals can be generated in the AOP, which is more highly toxic for different living beings [19,23].

Unlike the above studies, in this paper, the denitrifying respiratory process was efficient in completely mineralizing 10 mg E2-C L^−1^ and 9.09 mg NO_3_^−^-N L^−1^ to HCO_3_^−^ and N_2_, respectively, with efficiencies of 100% and yields close to 1.

### 3.5. Biodegradation of a Mixture of 17β-Estradiol and Acetate

**Figure 3:**
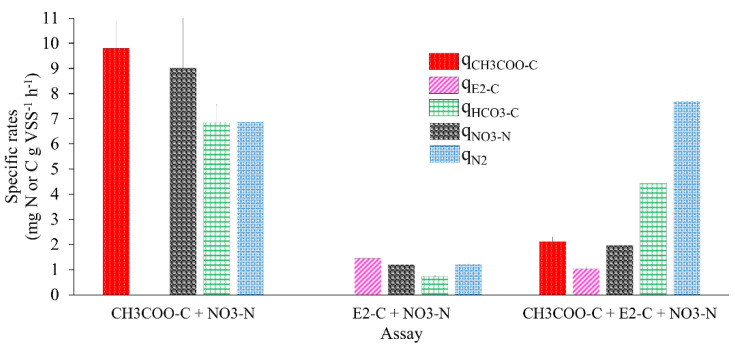
Specific rates (q) of substrate consumption and product generation in batch denitrifying assays.

The metabolic process of the denitrifying sludge in the presence of a mixture of 10 mg E2-C L^−1^ with 10 mg CH_3_COO^−^-C L^−1^ (Figure 3) showed an E_E2-C_, E_CH3COO_-_-C_, and E_NO3_-_-N_ of 100% (Table 2) at 25 h of reaction time.

Comparing the E2 and CH_3_COO^−^ consumption periods with those obtained in the assay with E2 as the only electron source and in the reference assay, they were 44 and 80% higher when using the mixture. Results were also higher than in the assays with E2, as the sole electron source and the reference assays were higher by 11 and 94%, respectively. 

It is worth noting that simultaneously with the oxidation of carbon sources, NO_3_^−^-N reduction occurred after 17 h of reaction. 

Results showed that the mixture of 17β-estradiol and CH_3_COO^−^ affected the metabolic process of the denitrifying sludge since a longer period was required for substrate consumption. In spite of this, the process was carried out efficiently.

**Figure 4:**
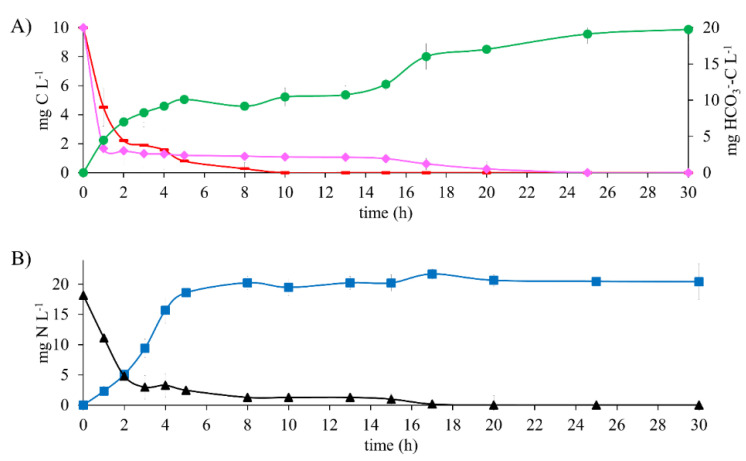
Kinetic profile of denitrifying assays with 10 mg E2-C L^−1^ in a mixture containing 10 mg CH_3_COO^−^-C L^−1^ at C N^−1^ of 1.1. (**A**) Carbon compounds: (♦) E2-C, (**▬**) CH_3_COO^−^-C, (●) HCO_3_^−^-C (secondary axis). (**B**) Nitrogen compounds: (■) N_2_, (▲) NO_3_^−^-N.

The kinetic profile of denitrifying assays (Figure 4A) shows that at 10 h of reaction time, the 10 mg CH_3_COO^−^-C L^−1^ is wholly consumed. In parallel, an average of 10 mg of HCO_3_^−^-C L^−1^ remained constant until 15 h of reaction time. Subsequently, a rise reached a maximum production of 19 mg HCO_3_^−^-C L^−1^, whereas the E2-C was consumed entirely. It is worth mentioning that when the mixture of CH_3_COO^−^-C and E2-C was tested, the total consumption of all substrates took 25 h. This is greater than the reference assays and tests with E2 as the sole electron source. Likewise, results show that CH_3_COO^−^-C was the first biodegraded carbon substrate. This is because it is considered an easily biodegradable compound [29]. Moreover, the inclusion of E2 did not generate any carbon or nitrogen intermediaries, despite working at a E2 to CH_3_COO^−^ ratio of 1:1. These results contrast with the report by Islas et al. [4], where 30 mg L^−1^ N-NO_2_^−^ was generated when testing a mixture of 10 mg C-AMP L^−1^ and 100 mg C-CH_3_COO^−^ L^−1^.

### 3.6. The Effect of E2 on the Kinetic Performance of the Denitrifying Sludge

Figure 3 and Table 2 show the kinetic performance of the denitrifying sludge in the reference assays with E2 as the sole carbon source compared to the mixture of E2 and CH_3_COO^−^.

When 10 mg CH_3_COO^−^-C L^−1^ was used, a q_CH3COO_^−^-_C_ of 9.79 ± 1.09 was obtained. With 10 mg E2-C L^−1^ as the only source of electrons, the q_E2-C_ was 1.44 ± 0.01, 5.79 times lower than the q_CH3COO_^−^-_C_ obtained in the reference assay; however, when tested with the mixture of E2 with CH_3_COO^−^, the q_C-E2_ obtained was 1.04 ± 0.08, 0.38 times lower than that obtained in the assay with E2 as the sole electron source. The q_CH3COO-_-_C_ was 2.12 ± 0.17, 3.61 times lower than that of the reference assay. Amin et al. [38] reported that when they studied E2 degradation using nitrifying bioreactors under a hydraulic retention time of up to 11 days, they obtained consumption rates of E2 and its metabolites, such as E1 and EE2, of 0.00141, 0.00116, and 0.00109 mg gr VSS^−1^ h^−1^, respectively. These were 1021.27, 1241.37, and 1321.10 times lower than the q_E2-C_ obtained in the tests with E2 as the sole electron donor. On the other hand, Peña et al. [30] studied the elimination of toluene and xylene by a denitrifying sludge, where they obtained rates up to 0.5 and 8 times lower than those of the assays with E2 as the sole electron donor. 

Figure 3 shows the kinetic behavior of the sludge with the mixture of E2 with CH_3_COO^−^ (q_NO3-N_, q_HCO3-C_, and q_N2_). The kinetic behavior was 0.63, 5.15, and 5.35 higher than in the assay, with E2 as a sole electron source. The specific rates were slightly lower than those achieved in the reference cultures. This proves that an alternate source of electrons that is easily biodegradable in the presence of a recalcitrant compound improves the kinetic performance of the denitrifying process. The q_E2-C_ calculated in the assay with the mixture of E2 and CH_3_COO^−^ was slightly lower than E2 as the only electron source. Despite this, the process was efficient.

Regarding the NO_3_^−^-N (q_NO3_^−^) consumption rate in the assay with E2-C as the sole electron source and in the mixture of E2 and CH_3_COO^−^, it was 6.5 and 3.5 times lower in relation to the q_NO3-_-_N_ obtained in the reference test with 10 mg of CH_3_COO^−^-C L^−1^ (Table 2), respectively. These results show that the presence of E2 as the sole electron source affected the NO_3_^−^ reduction rate. However, the presence of CH_3_COO^−^-C in the assay, when mixed with E2, improved the q_NO3-N_ by up to 0.63 times compared to the results obtained with E2 as the only electron source. These results coincide with studies that mention that the kinetic improvement of the respiratory process of a denitrifying sludge is found by adding an alternate source of electrons that is easily biodegradable, such as acetate, during the biodegradation of a recalcitrant compound. Such is the case in the work carried out by Martínez et al. [28], where they reported that the q_NO3-_-_N_ obtained in an assay with 22 mg 2-chlorophenol-C L^−1^ and 108 mg CH_3_COO^−^-C L^−1^ f was 2.7 times greater than the q_NO3-_-_N_ obtained in an assay with 22 mg 2-chlorophenol-C L^−1^ as the sole electron donor. Additionally, Martinez et al. [39] reported that q_NO3-N_ in assays with 2-chlorophenol but with readily biodegradable alternate electron sources, such as CH_3_COO^−^, glucose and phenol, improved by up to 1.4, 1.6, and 6 times.

The NO_3_^−^-N consumption rates were reported to be lower than the values in this study. For example, González et al. [40] reported a NO_2_^−^ consumption rate of 0.37 mg NO_2_^−^-N g VSS^−1^ h^−1^ with 50 mg *p*-cresol-C, and Martínez, et al. [28] reported a q_NO3-_-_N_ of 5.86 ± 0.01 mg NO_3_^−^-N g VSS^−1^ h^−1^ in assays with CH_3_COO^−^-C. However, some studies have investigated the denitrifying sludge behavior with some compounds considered as ECs [41]. In both studies, q_NO2-_-_N_ is 2.24 and 0.53 times higher than that reported here, with E2 as the sole electron donor and the reference assays. It is worth mentioning that, in the study by Martínez et al. [28], the assays used an easily biodegradable compound. 

In the case of the species produced in the denitrifying process, the q_HCO3-_-_C_ of 0.72 ± 0.02 and 4.43 ± 0.41 was obtained in assays with E2 as the sole electron source and the mixture of E2 and CH_3_COO^−^, being 8.5 and 0.54 times lower than the reference assays, respectively. The q_N2_ obtained in assays with E2 as the sole electron source was 4.6 times lower than in reference assays. When assays were carried out with the E2 and CH_3_COO^−^ mixture, q_N2_ improved; it was 0.11 and 5.34 times higher than the q_N2_ obtained in the reference assays and with the mixture of E2 and acetate. The results obtained from q_Substrates_ and q_Products_ indicated that the presence of E2 has a negative effect on the kinetics of the respiratory process of the denitrifying sludge. The above is attributed to the structure of E2 and its physical and chemical properties compared to acetate [21]. Despite this, the efficiencies were 100% in both cases, and product yields were greater than 0.9 (Table 2).

This is the first study demonstrating the final destination of 17β-estradiol in a denitrifying process by quantifying the production of HCO_3_^−^-C and N_2_ shown in the kinetic profiles. Furthermore, denitrification’s formation of intermediaries or secondary metabolites was not found in the assays, as indicated through the mass balance (Table 3). This contrasts with other reports where the final product of the process is not mentioned. [18,19,21,23,27,42]. These results demonstrate that there was no denitrifying enzyme inhibition due to the presence of E2. The respiratory process was efficient and effective for E2 biodegradation. Moreover, HCO_3_^−^ and N_2_ were the only products generated_._

The mass balance (Table 3) shows that substrate concentrations correspond exclusively to HCO_3_^−^-C and N_2_ [4,28]. It is also important to highlight that as the working ratio C N^−1^ was 1.1, it was close to the stoichiometric value. Biomass was not generated, and the E2 concentration was exclusively used to produce energy, but not for new cells. Finally, the possibility of the sludge entering into an endogenous regime in the presence of E2 as the sole electron source, as occurs in other studies with 2-chlorophenol [28], is ruled out by the response variables evaluated and the results obtained. 

## 4. Conclusions

The denitrifying sludge could biotransform up to 10 mg of E2-C L^−1^ with the consumption efficiencies of 100% for E2 and NO_3_^−^, showing yields of more than 0.9 in the generation of N_2_ and HCO_3_^−^ as the only products. Secondary metabolites such as E1, E3, N_2_O, and NO_2_^−^ were not generated.

Using 10 mg E2-C L^−1^ as the sole carbon source affected the kinetic and metabolic process of the denitrifying sludge in relation to the reference assay with 10 mg CH_3_COO^−^-C L^−1^. In spite of this, the process was efficient. 

With the use of 10 mg of CH_3_COO^−^-C L^−1^ as a co-substrate at a 1:1 proportion in E2 biodegradation, the respiratory sludge process improved with the assay where E2 was the sole electron source.

This is the first work where the response variables of the denitrifying process in the biotransformation of E2 as the only source of electrons and in the mixture with acetate are reported.

This study suggests that it is feasible to use a denitrification process in water contaminated with estradiol and some easily biodegradable compounds, such as acetate, for their elimination.

Therefore, this work provides the basic knowledge that can be used as a tool on a larger scale.

## Figures and Tables

**Table 1:** Processes for the treatment of water containing 17β-estradiol.

Compound (Concentration)	Process	Result	Reference
17β-Estradiol (E2) (1 µg L^−1^)	AOPs 100 mg L^−1^ of TiO_2_ Degussa P25 UV 400 W (254 nm)	E2 removal efficiency of 85% in 60 min. Possible free-radical formation. The oxidation product of E2 at the end of the process was not reported.	[23]
17β-Estradiol (E2) (10 µg L^−1^)	AOPs UV and UV/H_2_O_2_ Mercury lamp (1 kW)	Removal efficiency up to 90%. The oxidation products are unknown. Hydroxyl-radical formation (● OH) due to the interaction of UV light with H_2_O_2_.	[19]
17β-Estradiol (E2) (1 and 2 mg L^−1^)	Biomembrane system with *S. dimorphus* algae	E2 removal efficiency of 46.1%. E2 final destination was not reported. E2 and Cu (II) affected growth, chlorophyll, protein, and carbohydrate content in *S. dimorphus* after 12 days of culture.	[26]
17β-Estradiol (E2) (20 mg L^−1^) Tetracycline (0.1 mg L^−1^)	*Novosphingobium* sp. cells isolated from a WWTP sludge	E2 removal efficiency of 96% after 168 h. The final destination of the contaminant was not reported. The negative influence of tetracyclines on E2 degradation.	[17]
Ethinyl estradiol (30 mg L^−1^)	Evaluation with *Sphingobacterium* sp. JCR 5 (aerobic system)	Removal efficiency of 87% after 240 h of reaction. EE2 metabolite formation includes E1, 2-hydroxy-2,4-diene valeric acid, and 2-hydroxy-2,4-diene-1,6-dioic acid.	[18]
17β-Estradiol (E2) (5 mg L^−1^)	Culture evaluation with *Virgibacillus halotolerans*, *Bacillus flexus*, and *Bacillus licheniformis* (aerobic and anaerobic systems)	Biodegradation efficiency up to 80% in aerobic systems for 96 h, but with the formation of E1. *Bacillus licheniformis* biodegraded 68% of E2 in 2160 h of evaluation with 2 mg L^−1^ of estrone (E1) formation. No biotransformation products were reported.	[21]

**Table 2:** Response variables of the reference denitrifying assays, with estradiol (E2) as the only electron source and the assays with a mixture of E2 and CH_3_COO^−^.

Response Variable	CH_3_COO^−^-C + NO_3_^−^-N (Reference)		E2-C + NO_3_^−^-N		CH_3_COO^−^-C + E2-C + NO_3_^−^-N	
**E**_**CH3COO**_^**−**^_**-C**_	100 ± 0.01		-		100 ± 0.01	
**E**_**E2-C**_	-		100 ± 0.01		99.13 ± 1.49	
**E**_**NO3**_^**−**^_**-N**_	100 ± 0.01		100 ± 0.01		100 ± 0.01	
**Y**_**HCO3**_^**−**^_**-C**_	1.02 ± 0.01		0.91 ± 0.03		0.96 ± 0.02	
**Y**_**N2**_	1.06 ± 0.02		0.96 ± 0.02		1.12 ± 0.01	
**Linear regression coefficients**		R^2^		R^2^		R^2^
**q**_**CH3COO**_^**−**^_**-C**_	9.79 ± 1.09	0.9522	-	-	2.12 ± 0.17	0.9727
**q**_**E2-C**_	-	-	1.44 ± 0.01	0.9992	1.04 ± 0.08	0.9303
**q**_**NO3**_^**−**^_**-N**_	9.01 ± 2.40	0.9787	1.20 ± 0.02	0.9965	1.96 ± 0.16	0.9245
**q**_**HCO3**_^**−**^_**-C**_	6.86 ± 0.68	0.9265	0.72 ± 0.02	0.9522	4.43 ± 0.41	0.9028
**q**_**N2**_	6.88 ± 0.86	0.9406	1.21 ± 0.02	0.9860	7.69 ± 0.26	0.9808

E = [(mg consumed substrate)/(mg initial substrate)] × 100; Y = (mg generated product)/(mg consumed substrate); q = mg g VSS ^−1^ h^−1^.

**Table 3:** Mass balance of denitrifying assays.

Assay	Input (mg L^−1^)			Output (mg L^−1^)		
	CH_3_COO^−^-C	E2-C	NO_3_^−^-N	HCO_3_^−^-C	CO_2_-C	N_2_
**(1)**	10.00 ± 0.01	-	9.09 ± 0.01	10.19 ± 0.14	-	9.63 ± 0.18
**(2)**	-	10.00 ± 0.01	9.09 ± 0.01	9.07 ± 0.37	-	8.72 ± 0.05
**(3)**	10.00 ± 0.01	10.00 ± 0.01	18.18 ± 0.01	19.16 ± 0.56	-	20.52 ± 0.12

(1) Reference assay with 10 mg CH_3_COO^−^-C L^−1^ and 9.09 mg NO_3_^−^-N L^−1^; (2) 10 mg E2-C L^−1^ and 9.09 mg NO_3_^−^-N L^−1^; (3) 10 mg CH_3_COO^−^-C L^−1^, 10 mg E2-C L^−1^, and 18.18 mg NO_3_^−^-N L^−1^.

## Data Availability

Not applicable.
